# Early Postoperative Nausea and Vomiting After Bariatric Surgery: A Study of 8426 Patients from the Swedish Perioperative Registry (SPOR)

**DOI:** 10.1007/s11695-025-08351-0

**Published:** 2025-11-04

**Authors:** Jakob Walldén, Mattias Larsson, Antonio Moraitis, Sandra Ahlqvist, Yucel Cengiz, Tomi Myrberg, Helena Nyström, Magnus Hultin

**Affiliations:** 1 Department of Diagnostics and Intervention and Department of Anaesthesiology and Intensive Care (Sundsvall), Umeå University, Umeå, Sweden; 2 Department of Diagnostics and Intervention and Centre of Surgery (Sundsvall), Umeå University, Umeå, Sweden; 3 Department of Diagnostics and Intervention and Centre of Anaesthesiology and Intensive Care Medicine (Sunderbyn), Umeå University, Umeå, Sweden; 4 Department of Diagnostics and Intervention and Department of Anaesthesiology and Intensive Care (Umeå), Umeå University, Umeå, Sweden

**Keywords:** Postoperative nausea and vomiting, Bariatric surgery, Perioperative care, Patient outcomes

## Abstract

**Background:**

The reported incidence of postoperative nausea and vomiting (PONV) after laparoscopic bariatric surgery is up to 60–80%. Hower, studies are limited, and larger studies are warranted. As PONV is usually evaluated in the post-anesthesia care unit (PACU), studying early PONV can be a valuable tool for exploring risk and associated factors for PONV.

**Methods:**

Using prospectively collected data from the Swedish perioperative registry (SPOR) from 2016 to 2022, we explore the incidence and associated factors for early PONV after laparoscopic bariatric surgery. Laparoscopic gastric bypass and laparoscopic gastric sleeve procedures in adult patients (≥ 18 years) were included. The primary outcome was the incidence of PONV in the PACU. Secondary outcomes were factors associated with PONV, which were analyzed using a multivariate logistic regression model.

**Results:**

In total, 14,098 procedures were identified in the registry during the study period, and 8426 unique patients from 32 hospitals in Sweden were included in the final study cohort. PONV in PACU was present in 36% (*n* = 3018) of patients. Factors associated with early PONV were female sex, age, moderate-severe pain, gastric sleeve procedures, duration in PACU, and hospital.

**Conclusions:**

In this national register-based cohort study, one third of patients experienced early PONV in the PACU after laparoscopic bariatric surgery. Several risk factors were associated with increased occurrence of PONV, and there was variability among hospitals in the incidence of PONV.

Clinicaltrials.gov: NCT04433676

**Supplementary Information:**

The online version contains supplementary material available at 10.1007/s11695-025-08351-0.

## Introduction

After laparoscopic bariatric surgery, the incidence of postoperative nausea and vomiting (PONV) is high, reportedly being up to 60–80% [1–5]. Standard perioperative antiemetic guidelines are insufficient to prevent PONV events after bariatric surgery [6, 7], and PONV is considered an important cause of postoperative morbidity and prolonged stay [8, 9]. To improve patient care, it is important to identify factors associated with increased risk of PONV and to tailor specific antiemetic strategies. Furthermore, as studies assessing the incidence of PONV after bariatric procedures are limited, often being single-centre studies including small numbers of patients, larger studies are warranted [7].

Almost all hospitals in Sweden submit data to the Swedish Perioperative Registry (SPOR), which covers the perioperative process up to discharge from the post-anesthesia care unit (PACU). The registry includes evaluations of PONV in the PACU (early PONV), which might also be considered a proxy for overall PONV [10].

The aim of this study was to describe the incidence of early PONV after laparoscopic bariatric surgery using the Swedish national SPOR registry and to identify factors associated with early PONV.

## Materials and Methods

This observational national registry-based cohort study was based on prospectively collected perioperative data. We included laparoscopic bariatric procedures from the Swedish Perioperative Registry (SPOR) from 2016 to 2022. The study was approved by the Swedish Ethical Review Authority, Uppsala, Sweden on 20 March 2019 (chairperson E. Schön-Engqvist ref. number 2019-01522) and reported according to the STROBE Statement [11].

### Inclusion/Exclusion

Inclusion criteria were patients ≥ 18 years of age undergoing elective laparoscopic Roux-en-Y gastric bypass (LRYBG) or laparoscopic sleeve gastrectomy (LSG), assessed with PONV in the PACU and having an anaesthesia procedure code in the registry.

Exclusion criteria were multiple procedures in the same patient (only the first procedure was included).

### Data Sources

The SPOR-registry contains perioperative data from the majority of hospitals in Sweden. We included the following variables: hospital/unit, age, sex, body weight, body height, ASA-classification, smoking status, surgical procedure codes, anesthesia procedure codes, in/outpatient, duration of anesthesia and procedure, pain assessment in the PACU, presence of nausea or vomiting in the PACU, and time in the PACU including timestamps.

Data submitted to the registry are prospectively collected during patient care and automatically submitted to SPOR from the local hospital databases/registries.

The registry includes only data up to discharge from the PACU. Variables processed from the registry were patient, perioperative, and postoperative characteristics. The anesthesia code in the registry was used to extract anesthesia-related variables. A list of all variables and source of origin is available in Supplement 1.

The variable PONV in the PACU was registered and defined as the worst nausea/vomiting during the stay in the PACU (no nausea/nausea/vomiting). We defined the primary outcome variable (early PONV) as either nausea or vomiting in the PACU.

### Data Processing

The database from the SPOR registry was retrieved in August 2023 and processed and analyzed using the statistical software SPSS Statistics for Macintosh, Version 29, released 2023 (IBM Corp., Armonk, NY, USA) and R version 4.5.0 (R Core Team 2025).

### Statistical Analysis

Descriptive methods were used to present the data, and values are presented as numbers with percentages (%), means with standard deviations (SD) or median with interquartile ranges (IQR). To analyze factors associated with early PONV, univariate analyses were performed using Pearson’s chi-squared test and presented with odds ratios (ORs), 95% confidence intervals, and *p*-values.

To compare the incidences of PONV between hospitals, we employed the one-sample proportion test, allowing us to describe whether differences existed between the observed proportions of PONV in each hospital and the overall incidence of PONV. The results are presented visually with incidences, including the 95% confidence intervals for the proportions.

A multivariate logistic regression analysis was performed. We decided to include two models in the report, one including and one excluding hospital as a factor in the model. The reference hospital in the multivariate analysis was determined by ranking all hospitals based on the incidence of PONV and selecting the hospital that divided the study cohort into two equal halves in terms of number of patients.

We included all relevant variables in the models, and variables with the highest *p*-values were excluded stepwise one by one if *p* > 0.10. At each step, patients with missing data in the variables under consideration were excluded from that specific model iteration but could be re-included in subsequent steps if the relevant variables were no longer part of the model. The final models are presented with adjusted odds ratios (aORs) and the corresponding 95% confidence intervals and *p*-values.

## Results

**Fig. 1:**
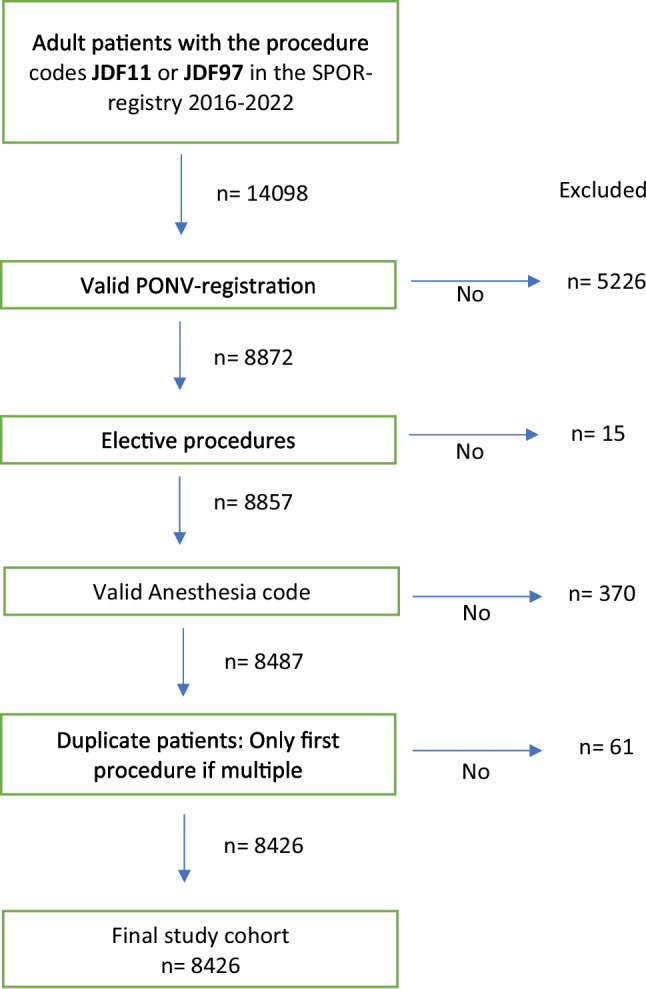
Flow-chart for the selection of the study cohort

We identified 14,098 patients in the registry from 2016 to 2022 who had eligible procedures; after applying the inclusion and exclusion criteria, the final study cohort consisted of 8426 patients. The main reasons for exclusion were a missing registration for PONV in the PACU and a missing code for the anesthesia method (Fig. 1).

In our cohort, 75% (*n* = 6344) were females and the mean age was 42 (12) years. The mean body mass index (BMI) was 41 (5.6) kg·m^–2^, and the majority of patients, 64% (*n* = 5417), underwent laparoscopic Roux-en-Y gastric bypass (LRYGB). Volatile anesthesia was the main anesthesia method used (72%, *n* = 6041). Further characteristics are presented in Table 1.

**Table 1:** Characteristics of patients in the study cohort; values are numbers (%) or means (SD)

**Variable**	**Value**	**Missing values**
Sex, female	6344 (75%)	15
Age, years	42 (12)	12
BMI, kg m^–2^	41 (5.6)	3077
Smokers	166 (7%)	6040
ASA classification		119
1	388 (5%)	
2	4036 (49%)	
3	3862 (46%)	
4	21 (0%)	
Surgical procedure		0
LSG	3008 (36%)	
LRYGB	5418 (64%)	
Duration of surgery, minutes	70 (30)	0
Main anaesthesia method		4
Volatile anaesthesia	6041 (72%)	
Total intravenous anaesthesia	2381 (28%)	

LSG = laparoscopic sleeve gastrectomy; LRYGB = laparoscopic Roux-en-Y gastric bypass.

PONV in the PACU was present in 36% (*n* = 3018) of the patients. The median time in the PACU was 170 min, and 40% of patients (*n* = 3310) experienced severe pain (NRS > 5) in the PACU (Table 2).

**Table 2:** Outcome measures in the post-anaesthesia care unit (PACU); values are numbers (%), means (SD) or medians (IQR)

Variable	Value	Missing values
Postoperative nausea or vomiting (PONV)	3018 (36%)	0
Maximal pain in PACU (NRS)		250
Mean	4.4 (3)	
Median	5 (2–7)	
Moderate to severe pain in PACU (NRS > 5)	3310 (40%)	250
Duration in PACU, minutes		0
Mean	212 (191)	
Median	170 (127–232)	

SD = standard deviation; IQR = interquartile range, 25%–75% percentile

NRS = numerical rating scale; PACU = post-anaesthesia care unit.

The univariate analysis of factors associated with PONV is presented in Table 3. Female sex (OR 2.12 [1.89–2.37]; *p* < 0.001), moderate to severe pain (OR 1.73 [1.58–1.89]; *p* < 0.001), and laparoscopic sleeve gastrectomy (OR 1.60 [1.46–1.75]; *p* < 0.001) were the strongest associated factors.

**Table 3:** Univariate analysis of factors associated with early postoperative nausea or vomiting (PONV) after laparoscopic bariatric procedures

Variable	Category	Number (%)	Risk of PONV	Univariate analysis	
				OR (95% CI)	*p*-value
Sex	*Female*	6344 (75%)	39.7%	2.12 (1.89–2.37)	<0.001
	*Male*	2067 (25%)	23.8%		
Age	≤*40 years*	4046 (48%)	40.5%	1.49 (1.36–1.62)	<0.001
	*>40 years*	4368 (52%)	31.4%		
BMI	≤*40*	2581 (48%)	37.5%	1.11 (1.00–1.24)	0.057
	*>40*	2768 (52%)	35.0%		
Smoking status	*Non-smoker*	2220 (93%)	35.9%	1.01 (0.73–1.40)	0.950
	*Smoker*	166 (7%)	36.1%		
ASA class	*1–2*	4424 (53%)	38.6%	1.30 (1.19–1.42)	<0.001
	*3–4*	3883 (47%)	32.7%		
Surgical method	*LSG*	3008 (36%)	42.8%	1.60 (1.46–1.75)	<0.001
	*LRYGB*	*5418* (64%)	31.9%		
Anaesthetic method	*TIVA*	2381 (28%)	37.3%	1.10 (0.99–1.22)	0.071
	*Volatile*	6041 (72%)	35.2%		
Duration of surgery	*<60 min*	3449 (41%)	37.8%	1.15 (1.05–1.26)	0.002
	*≥60 min*	4977 (59%)	34.5%		
Duration in PACU	*<180 min*	4609 (55%)	35.6%	1.02 (0.93–1.11)	0.687
	*≥180 min*	3817 (45%)	36.0%		
Moderate to severe pain in PACU	*>NRS 5*	3310 (40%)	43.6%	1.73 (1.58–1.89)	<0.001
	≤*NRS 5*	4866 (60%)	30.9%		

NRS = numerical rating scale, LSG = laparoscopic sleeve gastrectomy, LRYGB = laparoscopic Roux-en-Y gastric bypass, TIVA = total intravenous anaesthesia, PACU = post-anaesthesia care unit, OR = odds ratio, CI = confidence interval.

**Fig. 2:**
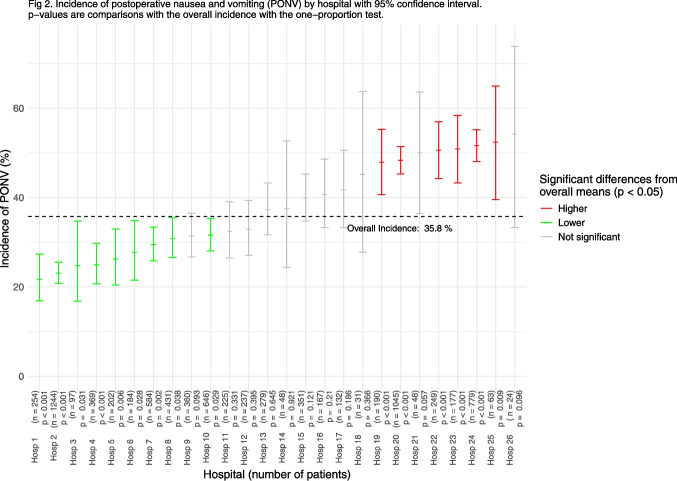
Incidence of postoperative nausea and vomiting (PONV) by hospital with 95% confidence interval. p − values are comparisons with the overall incidence with the one − proportion test

The overall incidence of PONV varied among the hospitals, ranging from 22 to 54% (see Fig. 2 and Supplement 2).

The final multivariate model (including hospital as a factor) identified several independent significant factors associated with PONV risk: female sex (aOR 2.21 [1.96–2.50]; *p* < 0.001), age (aOR 0.85 [0.81–0.88]; *p* < 0.001), laparoscopic sleeve gastrectomy (aOR 1.69 [1.51–1.89]; *p* < 0.001), moderate to severe pain in the PACU (aOR 1.56 [1.41–1.72]; *p* < 0.001), time in the PACU (aOR 1.03 1.01–1.06]; *p* = 0.003), and hospital (*p* < 0.001) (Table 4).

**Table 4:** Multivariate logistic regression model of independent factors associated with postoperative nausea or vomiting (PONV) in the PACU (n = 8158)

Variable	Adjusted OR (95% CI)	*p*-value
Sex (female)	2.21 (1.96–2.50)	<0.001
Pain >NRS 5 in PACU	1.56 (1.41–1.72)	<0.001
Laparoscopic sleeve gastrectomy (LSG)	1.69 (1.51–1.89)	<0.001
Age, per decade	0.85 (0.81–0.88)	<0.001
Duration in PACU (per hour)	1.03 (1.01–1.06)	0.003
Hospital No.		<0.001
3	0.47 (0.23–0.94)	0.034
5	0.70 (0.48–1.02)	0.066
1	0.71 (0.50–1.02)	0.061
2	0.81 (0.65–1.02)	0.068
7	0.81 (0.63–1.05)	0.116
12	0.90 (0.64–1.26)	0.530
4	0.91 (0.67–1.24)	0.565
8	0.94 (0.71–1.23)	0.634
10	*Reference*	
16	1.02 (0.70–1.48)	0.912
6	1.04 (0.71–1.53)	0.838
9	1.09 (0.82–1.46)	0.548
11	1.0 (0.79–1.54)	0.581
13	1.50 (1.1–2.05)	0.011
17	1.53 (1.03–2.27)	0.035
19	1.60 (1.13–2.26)	0.008
25	1.60 (0.93–2.77)	0.092
22	1.65 (1.21–2.25)	0.002
21	1.70 (0.92–3.16)	0.093
14	1.76 (0.94–3.29)	0.075
15	1.78 (1.34–2.36)	<0.001
24	2.43 (1.92–3.07)	<0.001
20	2.62 (2.11–3.26)	<0.001
23	2.64 (1.85–3.75)	<0.001
18	2.78 (1.31–5.92)	0.008
26	2.99 (1.21–7.36)	0.018

PACU = post-anaesthesia care unit, NRS = numerical rating scale.

Cases at hospitals with < 5 cases were excluded from this multivariate analysis (5 hospitals with only one case.).

Variables entered in the logistic regression model: *Categorical:* gender, ASA class, surgical method, anaesthetic method, severe pain in PACU, and hospital. *Continuous:* age (per decade), body mass index (BMI), duration of surgery (per hour), and duration in PACU (per hour). Variables with the highest *p*-values were excluded stepwise one by one if *p* > 0.10. Cases with missing variables were excluded from the analysis.

The numbering of hospitals based in incidences of PONV (see Supplement 2). The reference hospital was selected as the one, according to this order, that included the 4263rd patient (the midpoint of the study cohort).

In the alternative multivariate model, not including hospital as a factor, independent significant factors were female sex (aOR 2.06 [1.83–2.32]; *p* < 0.001), age (aOR 0.85 [0.81–0.88]; *p* < 0.001), laparoscopic sleeve gastrectomy (aOR 1.54 [1.40–1.70]; *p* < 0.001), moderate to severe pain (aOR 1.61 [1.47–1.77]; *p* < 0.001), and ASA class (aOR 1.16 [1.05–1.27]; *p* < 0.003) (Supplement 3).

## Discussion

Using a national registry, we analyzed the incidence of early PONV after laparoscopic bariatric surgery in 8426 patients. We found that over one third of patients experienced PONV during early recovery and could identify and confirm several known risk factors for PONV.

Our reported incidence of early PONV (36%) is in line with that identified in previous studies [1, 12, 13]. Although numerous studies have reported high incidences of PONV after bariatric surgery, most such studies are single-center studies including a limited number of patients. The present study encompasses patients from 32 Swedish hospitals over a seven-year period and confirms the very high early PONV risk after bariatric surgery.

As previously shown, female sex and younger age were associated with a higher risk of PONV. The magnitudes of these risks align with previously reported risks from observational studies in diverse surgical cohorts [14]. PONV was also associated with moderate to high pain scores in the PACU. Postoperative pain is a factor that can be remediated clinically. The registry data from SPOR do not provide any data on the administered analgesics, so we cannot draw any conclusions as to the choice of specific analgesic strategies.

Total intravenous anesthesia (TIVA) is usually associated with lower risks of PONV compared with volatile anesthesia, but we found no such association. Generally, volatile agents increase the risk of PONV during the first postoperative hours [15], and a recent meta-analysis found benefits of using TIVA in bariatric procedures [16, 17]. However, adding extra PONV prophylaxis to volatile anesthesia might compensate for these differences [18]. Anesthetic technique is usually part of the standardized procedures at each hospital and is tightly linked to overall perioperative care, including surgical techniques and PONV prophylactic regimes. This might explain why we did not find any association between anesthetic regime and PONV.

Our results indicate an association between PONV and prolonged PACU stay; however, this relationship is likely driven by patients requiring treatment and extended recovery due to PONV. This suggests that PONV contribute to longer PACU duration, rather than resulting for it.

Interestingly, there were major differences among the hospitals in PONV risk, which ranged from 22 to 54%, and different routines and thresholds for registering PONV events during PACU care might have contributed to this. However, as there might be real differences in PONV risk, we hypothesize that some hospitals have a more effective perioperative protocol to address PONV. We can only speculate whether this involves anesthetic techniques, more aggressive PONV prophylaxis, broad-spectrum multimodal analgesia, or other unknown factors. Understanding what constitutes the more effective perioperative protocols and implementing this knowledge clinically may reduce the PONV risk for many patients.

There are several possible explanations for the high risk of PONV in laparoscopic bariatric surgery. The surgical trauma per se might trigger PONV, as the stomach and vagus nerve are involved in the systems controlling nausea and vomiting [19]. Furthermore, there are differences between the surgical techniques in how the vagal nerve is damaged. In LSG, the peripheral branch is more affected, while in LRYGB the vagal nerve might be damaged on a truncal level [20]. We can only hypothesize whether this contributes to the differences in PONV observed between the techniques.

Although we used a national perioperative registry, we were unable to include all patients undergoing laparoscopic bariatric surgery in Sweden during the study period. According to open reports from the national registry for bariatric surgery [21], approximately 28,000 bariatric procedures were performed during the studied years. However, SPOR does not have full coverage, and it was only recently that all public hospitals in Sweden joined the SPOR registry; many private health providers still do not submit data to SPOR.

We identified 14,098 eligible patients in SPOR, but our final cohort consisted of 8426 patients. The main reason for exclusion was patients at hospitals not submitting the PONV variable. This was a pre-known limitation, as some hospitals did/do not collect and/or submit postoperative outcomes from the PACU to SPOR. In 2016, only 43% of procedures in our cohort had a valid PONV variable, a proportion increasing to 83% in 2022. However, these missing data are not random. For hospitals submitting the postoperative variables, prospective registration during patient care is compulsory. Therefore, patients in our final cohort are in a consecutive series of all bariatric procedures at each included hospital.

A further limitation of the study is the composition of the primary outcome variable (PONV yes/no). With this dichotomous outcome, we have no information about the extent to which patients were affected by PONV; however, previous reports have shown that around half of the patients with PONV during the first 24 h after bariatric surgery hours had severe nausea [1]. If quality-of-recovery (QoR) scales are included in outcome measures, several domains of recovery can be assessed and add quality to studies [22]. However, early PONV has recently been identified as one of eight important endpoints in clinical trials intended to improve postoperative patient comfort [23].

Also, an important limitation of this study is the lack of data in SPOR on intraoperative and postoperative opioid and antiemetic administration, as well as the implementation of Enhanced Recovery After Bariatric Surgery (ERABS) protocols across participating clinical sites. These anesthesia-specific variables are known to significantly influence the incidence of PONV and remain unaccounted for.

Administering adequate PONV prophylaxis is one of the cornerstones of managing PONV [6]. The high incidences call into question the use of standard algorithms for PONV in bariatric surgery [2, 7]. With extended multimodal PONV prophylaxis including NK-1 receptor antagonists, or by using opioid-sparing/opioid-free anesthesia, the PONV risk might be further reduced [12, 24–26].

Finally, our findings underscore the potential value of a national registry for monitoring the effectiveness of clinical pathways. To enable meaningful comparisons across institutions, such a registry in this context should incorporate detailed information on the protocols in use, including anesthesia techniques, analgesic and antiemetic regimens, and recovery models. This would facilitate evidence-based refinements in perioperative care and support improved patient outcomes at a national level.

## Conclusion

PONV risk is very high after laparoscopic bariatric procedures. One third of patients experienced early PONV, and several risk factors for PONV contribute to the observed early PONV. Novel strategies must be evaluated and implemented, as standard PONV algorithms are insufficient. We also suggest future studies to explore the mechanistic properties driving the development of PONV in this group.

## Supplementary Information

Below is the link to the electronic supplementary material.

Supplementary file1 (XLSX 12 KB)

Supplementary file2 (DOCX 40 KB)

Supplementary file3 (DOCX 35 KB)

## Data Availability

The dataset provided from the SPOR-registry are not publicly available. Upon reasonable request, the dataset might be shared after additional approvals from the SPOR-registry and the Swedish Ethical Review Board.

## Declarations

### Disclaimer

The data presented in this study were drawn from the Swedish Perioperative Register (SPOR). SPOR does not take responsibility for the methods, analysis, and results, and the views expressed in this study may not necessarily reflect those of SPOR.
