# Supplementary material for: Early Postoperative Nausea and Vomiting After Bariatric Surgery: A Study of 8426 Patients from the Swedish Perioperative Registry (SPOR)
Source: Obes Surg. 2025 Nov 4;35(12):5308–15. doi: 10.1007/s11695-025-08351-0 (PMC12722411; doi:10.1007/s11695-025-08351-0)
Supplement: Supplementary file 2 — Supplementary file2 (DOCX 40 KB) [file 11695_2025_8351_MOESM2_ESM.docx]

**Supplement 2** Incidences of early PONV and mean time in the PACU after laparoscopic bariatric surgery at each hospital in the study cohort, ordered from the lowest to highest overall incidences of PONV.

**All procedures LSG LRYGB**

**Hospital Incidence of PONV PACU time Incidence of PONV PACU time**  **Incidence of PONV PACU time**

Number % minutes Number % minutes Number % minutes

1 55/254 21.7% 195 (126) 0/0 NA NA 55/254 21.7% 195 (126)

2 287/1244 23.1% 160 (82) 78/228 34.2% 165 (56) 209/1016 20.6% 159 (86)

3 24/97 24.7% 1371 (132) 6/13 46.2% 1376 (123) 18/84 21.4% 1370 (135)

4 92/369 24.9% 202 (69) 59/196 30.1% 207 (74) 33/173 19.1% 196 (64)

5 53/202 26.2% 250 (117) 36/113 31.9% 256 (146) 17/89 19.1% 242 (64)

6 51/184 27.7% 314 (183) 1/1 100.0% 307 (0) 50/183 27.3% 314 (184)

7 172/584 29.5% 328 (190) 103/309 33.3% 342 (199) 69/275 25.1% 312 (178)

8 133/431 30.9% 177 (88) 78/255 30.6% 182 (108) 55/176 31.3% 170 (46)

9 113/360 31.4% 192 (82) 39/108 36.1% 189 (90) 74/252 29.4% 193 (78)

10 204/646 31.6% 194 (183) 120/294 40.8% 203 (184) 84/352 23.9% 186 (182)

11 73/225 32.4% 184 (73) 22/51 43.1% 215 (114) 51/174 29.3% 175 (54)

12 78/237 32.9% 128 (44) 51/133 38.3% 126 (42) 27/104 26.0% 130 (46)

13 104/279 37.3% 256 (137) 12/22 54.5% 252 (90) 92/257 35.8% 256 (140)

14 18/48 37.5% 252 (74) 5/8 62.5% 275 (67) 13/40 32.5% 247 (75)

15 140/351 39.9% 229 (112) 54/122 44.3% 230 (129) 86/229 37.6% 214 (103)

16 68/167 40.7% 368 (352) 67/160 41.9% 350 (334) 1/7 14.3% 782 (513)

17 55/132 41.7% 198 (74) 26/47 55.3% 203 (59) 29/85 34.1% 195 (82)

18 14/31 45.2% 230 (102) 3/5 60.0% 214 (74) 11/26 42.3% 237 (107)

19 91/190 47.9% 264 (105) 67/128 52.3% 264 (85) 24/62 38.7% 264 (137)

20 505/1045 48.3% 137 (78) 75/123 61.0% 137 (84) 430/922 46.6% 137 (78)

21 24/48 50.0% 226 (84) 16/31 51.6% 228 (98) 8/17 47.1% 221 (50)

22 126/249 50.6% 269 (197) 116/227 51.1% 252 (142) 10/22 45.5% 445 (457)

23 90/177 50.8% 127 (19) 42/67 62.7% 132 (22) 48/110 43.6% 124 (17)

24 402/779 51.6% 127 (48) 173/285 60.7% 126 (59) 229/494 46.4% 129 (106)

25 33/63 52.4% 405 (148) 33/62 53.2% 389 (67) 0/1 0.0% 1439 (NA)

26 13/24 54.2% 400 (369) 6/13 46.2% 363 (294) 7/11 63.6% 443 (454)

(Hospitals with limited number of cases)

27 0/1 0% 180 (NA) 0/1 0% 180 (NA) 0/0 NA NA

28 0/1 0% 212(NA) 0/1 0% 212 (NA) 0/0 NA NA

29 0/1 0% 1386 (NA) 0/0 NA NA 0/1 0% 1386 (NA)

30 0/1 0% 362 (NA) 0/0 NA NA 0/1 0% 362 (NA)

31 0/5 0% 512 (127) 0/5 0% 512 (127) 0/0 NA NA

32 0/1 0% 215 (NA) 0/0 NA NA 0/1 0% 215 (NA)

**Total** 3018/8426 **35.8%** 211 (189) 1288/3008 **42.8%** 226 (177) 1730/5418 **31.9%** 204 (197)

LGS = laparoscopic sleeve gastrectomy, LRYGB = laparoscopic Roux-en-Y gastric bypass, PONV = postoperative nausea and vomiting,
PACU = post-anaesthesia care unit, NA = not applicable.
Values are numbers, percentages (%), or means with standard deviations (SD).
