# Supplementary material for: Early Postoperative Nausea and Vomiting After Bariatric Surgery: A Study of 8426 Patients from the Swedish Perioperative Registry (SPOR)
Source: Obes Surg. 2025 Nov 4;35(12):5308–15. doi: 10.1007/s11695-025-08351-0 (PMC12722411; doi:10.1007/s11695-025-08351-0)
Supplement: Supplementary file 3 — Supplementary file3 (DOCX 35 KB) [file 11695_2025_8351_MOESM3_ESM.docx]

### **Supplement 3** Multivariate logistic regression model of independent factors associated with postoperative nausea or vomiting (PONV) in the PACU after laparoscopic bariatric surgery (n = 8048) without including hospital as a factor.

**Variable Adjusted OR (95% CI) *p*-value**

Sex (female) 2.06 (1.83–2.32) <0.001

Pain >NRS 5 in PACU 1.61 (1.47–1.77) <0.001

Laparoscopic sleeve gastrectomy (LSG) 1.54 (1.40–1.70) <0.001

Age, per decade 0.85 (0.81–0.88) <0.001

ASA class 1–2 1.16 (1.05–1.27) 0.003

PACU = post-anaesthesia care unit, NRS = numerical rating scale.

Variables entered in the logistic regression model: *Categorical:* gender, ASA class, surgical method, anaesthetic method, and severe pain in PACU. *Continuous:* age (per decade), body mass index (BMI), duration of surgery (per hour), and duration in PACU (per hour). Variables with the highest *p*-values were excluded stepwise one by one if *p* > 0.10.
